# Survey of junior doctors' perspective of serious incident reviews

**DOI:** 10.1192/bjo.2021.424

**Published:** 2021-06-18

**Authors:** Olusegun Popoola, Kuben Naidoo, Amrith Shetty

**Affiliations:** 1 Mersey Care NHS Foundation Trust; 2 Cheshire and Wirral Partnership NHS Foundation Trust

## Abstract

**Aims:**

Serious incidents according to NHS England (2015) are incidents where the consequences to patients, families and carers, staff or organisations are so significant or potential for learning are so great that a heightened response is justified. There is anectoctal evidence that this process is potentially difficult for junior doctors and the primary purpose of learning may be lost due to the stress involved.

Our aim was to evaluate junior doctors perspective of serious incident reviews. A secondary aim was to organise local and regional workshops based on the outcome of our findings to address misconceptions around serious incident investigations.

**Method:**

A survey was developed using survey monkey and distributed to all trainees across the Mersey region through the Medical Education teams.

The junior doctors range from core trainees to higher trainees. The survey encouraged the use of free texting if necessary.

Results from the survey were then analysed

**Result:**

18 junior doctors across the 3 mental health Trusts in the Mersey region responded.

12 respondents have been involved in a serious incident investigation in the past and 9 of the respondents stated that they did not recieve any support during the process. Out of the 3 that were supported, one rated the support as poor and frightening.

55.56% af all respondents found the process of serious incident reviews hard to understand.

66% of all respondents admitted that they are aware that the purpose of the review is for learning purposes.

100% of respondents agreed that a workshop to discuss the purpose and process of serious incidents investigation to aid their understanding would be useful.

**Conclusion:**

From the survey, we concluded that junior doctors do have some understanding of incident reviews process but they still do not feel comfortable with the idea of being under ‘investigation'.

It is also important that formal support is made available during the process.

We organised a workshop in one of the 3 Trusts which was well attended and junior doctors asked if they could sit on review panels for experiential learning. This is to be presented to govenance teams across the mental health trusts in the region.

Further workshop across the 2 remaining Trusts could not be organised due to COVID-19 pandemic.

